# Peripartum issues in the inflammatory arthritis patient: A survey of the RAPPORT registry

**DOI:** 10.1038/s41598-020-60451-2

**Published:** 2020-02-28

**Authors:** T. D. Dissanayake, W. P. Maksymowych, S. O. Keeling

**Affiliations:** 1 Garneau Rheumatology, Unit 430, 11044-82 Ave Edmonton, Alberta, T6G 0T2 Canada; 2 Division of Rheumatology, Department of Medicine, Faculty of Medicine & Dentistry, University of Alberta, Alberta, Canada

**Keywords:** Patient education, Rheumatoid arthritis

## Abstract

Childbearing women with rheumatoid (RA) and psoriatic arthritis (PsA) have significant peripartum issues. A retrospective anonymous RedCAP survey of peripartum period in females with RA/PsA in the RAPPORT registry was performed. Completed analyses included descriptive statistics, Chi-square and Fisher’s exact test. 162 patients (133 RA/29 PsA) completed the survey (103 women having 234 pregnancies), 164 pregnancies occurring before and 70 pregnancies occurring after diagnosis. Pregnancy outcomes from 103 patients included: 96% live births, 1.9% stillbirths, 23% miscarriages, and 15% therapeutic abortions. A third of patients had fewer children than desired due to disease activity, medications and other reasons. For 63 pregnancies after diagnosis: (1) 49% of pregnancies received pre-conception counseling; (2) 65% described good disease control during pregnancy but 74% flared in the first 3 months postpartum; (3) 79% of pregnancies discontinued IA medications; (4) 35% of pregnancies occurred on biologic therapy at or prior to conception. Gestational age at time of delivery was 37–40 weeks in 58% (33/57) post-arthritis vs 66% (83/126) pre-arthritis pregnancies. No statistically significant differences occurred between pregnancies before or after RA/PsA diagnosis for: pregnancy planning, fertility treatment, pregnancy and labour/delivery complications, birth defect frequency or neonatal complications. Neonatal ICU admissions were significantly lower in pre- compared to post-arthritis pregnancies (3.2% vs 14.5%). No pregnancy complications were noted in 24/54 pregnancies on medications compared to 6/9 pregnancies not on medications. The impact of RA/PsA before, during and after pregnancy varied considerably in this cohort emphasizing the importance of informed-decision making at all stages.

## Introduction

Rheumatoid Arthritis (RA) and Psoriatic Arthritis (PsA) are two most frequent types of chronic systemic inflammatory arthritides (IA) that can cause irreversible joint damage, disability and increased mortality if sub-optimally treated^1–3^. Many studies have shown that early aggressive treatment of IA with disease modifying anti rheumatic drugs (DMARDs), which can be conventional synthetic DMARDs (csDMARDs), targeted synthetic DMARDs (tsDMARDs) and biologic DMARDs (bDMARDs), directed at achieving disease remission leads to better outcomes including reduction in pain, joint destruction, improved function, quality of life, and radiographic progression^4–7^.

For women of childbearing age with a diagnosis of RA/PsA, there are multiple issues surrounding the peri-partum period including medication use, fertility, risk of disease flare and potential impact on neonatal and maternal outcomes. We define peripartum period in this study as the immediate 3–6 month period preceding conception, time period during pregnancy and up to 24 months post partum. Furthermore, decision-making regarding medications before, during and after pregnancy are challenging, and can vary significantly between rheumatologists leading to great differences in the patient experience^8,9^.

Evidence about the peri-partum period in RA/PsA is limited to cohort studies with small numbers. In general, RA is seen to improve or remit in about 60–80% of patients during pregnancy; however, a significant number of patients still experience active disease, which require treatment decisions by the rheumatologist^10,11^. Therefore, some RA/PsA patients will choose to have smaller families (fecundity) to avoid the risk of flare during conception attempts or an unsuccessful pregnancy^10,12^. While certain DMARDs must be clearly discontinued due to teratogenic potential (eg. methotrexate), recommendations for the use of biologics during pregnancy emphasize that the decision should be individualized, although there is increasing evidence indicating safety of biologics during pregnancy^13^. Furthermore, the recommendation to discontinue certain TNF inhibitors (TNFi) at 20–32 weeks of pregnancy also lacks sufficient data^8,14^.

Considering all the challenging issues surrounding potential pregnancies in RA/PsA patients, the objectives of this study were: (1) To gain insight into fertility and fecundity in RA/PsA patients in northern Alberta participating in the Rheumatoid Arthritis Pharmacovigilance Program and Outcomes Research in Therapeutics (RAPPORT) prospective cohort (2) To describe the peripartum period for RA/PsA patients in northern Alberta interms of preconception counselling, unplanned/planned pregnancy, time to pregnancy, requirement for infertility treatment, pregnancy outcomes, number of pregnancies prior to and after IA diagnosis, disease activity during and after pregnancy, type of delivery and breast feeding (3) To understand the choices made by females with RA/PsA in childbearing age regarding arthritis medication usage (csDMARDs, tsDMARDs, bDMARDs, steroids, non-steroidal anti-inflammatories) (4) To understand the fetal and maternal outcomes in the peri-partum period for RA/PsA patients in northern Alberta in terms of neonatal complications, labour and delivery complications. We hypothesized that the presence of a RA/PsA diagnosis in women of childbearing age had important consequence on pregnancy, including decreased fecundity in patients after the diagnosis due to fear of adverse pregnancy outcomes as a result of the disease itself and medications, increased pregnancy complications in patients with active RA/PsA disease and treatment discontinuation.

## Materials and Methods

We conducted a survey-based retrospective evaluation of the peri-partum period in RA/PsA patients participating in the RAPPORT (Rheumatoid Arthritis Pharmacovigilance Program and Outcomes Research in Therapeutics) prospective cohort in northern Alberta, Canada. The RAPPORT program is an ongoing prospective inception cohort of IA patients on biologics in the province of Alberta, Canada since 2004. Total number of patients seen at the time of the survey was 2659. Following ethics approval from the University of Alberta Research Ethics Board (Pro00064218), an anonymous electronic-based survey was sent by email link to all female patients of child-bearing age (<50 years) in the RAPPORT database (440 patients in total) who have previously provided informed consented to participate in the RAPPORT program and agreed to receive communication regarding future studies and questionnaires related to RAPPORT. Implied consent was granted by the action of completing the survey sent by email link. All methods were performed in accordance with relevant guidelines and regulations. No paper-based version of the questionnaire was utilized. No authors had competing interests related to this study.

This survey collected the following information from the patients: (1) the impact of the disease on fertility and fecundity, pregnancy and post-partum period, (2) course of disease during pregnancy assessed based on whether joint pain, swelling or tenderness was limited during pregnancy, (3) complications of pregnancy including Preterm labour (delivery <37 weeks), Premature rupture of membrane, Prolonged labour (labour lasting for >20 hours (1st pregnancy) and >14 hours (subsequent pregnancies), Haemorrhage (bleeding), Infection, Umbilical cord prolapse (umbilical cord comes out of the uterus with or before the presenting part of the fetus), Stillbirth (baby born with no signs of life at or after 24 weeks gestation) (4) medication use during pregnancy including medications taken when the patient realized she was pregnant, medications discontinued and continued during pregnancy and biologic therapies, (5) post-pregnancy issues for the mother and baby including disease flare, breastfeeding, and medication changes, birth defects, neonatal ICU admission as well as (6) choices women make around pregnancy in general (Supplemental Appendix 1: Survey).

All information was collected online through RedCap, a web-based survey tool, which is supported through EPICORE (Epidemiology Coordinating and Research Centre) at the University of Alberta. All responses were obtained and collected over a 6-week period with email reminders sent twice over the period for those who did not respond. Information obtained through the survey was evaluated for pregnancy-related outcomes using descriptive statistics, Chi-Square and Fisher’s exact data. Chi-square and Fisher’s exact tests were used to analyze pregnancy variables such as pregnancy complications, pregnancy planning, labour and delivery complications in pre and post RA/PsA pregnancies.

## Results

During the 6-week collection period, 162 patients completed the survey (response rate of 37%), with 133 patients having a diagnosis of RA and 29 patients having a diagnosis of PsA, the majority of patients ranging in age from 41–50 (Table 1). One hundred and three (103) patients had 234 pregnancies with 164 (out of 234) pregnancies occurring prior to the RA/PsA diagnosis and 70 (out of 234) pregnancies occurring after the diagnosis. Seventeen patients had a pregnancy, developed RA/PsA and then had at least one more pregnancy after their diagnosis. Pregnancy outcomes from 103 patients included: 96% live births, 1.9% stillbirths, 23% miscarriages, and 15% therapeutic abortions (Table 1).

**Table 1:** Characteristics of RAPPORT Survey Patients.

		N (%)
Type of IA	RA	133 (82.1)
	PsA	29 (17.8)
Age Range	<19 years	1 (0.6)
	20–30 years	17 (10.5)
	31–40 years	55 (34)
	41–50 years	89 (36.4)
Antibodies positivity	RF+	59 (36.4)
	Anti-CCP+	6 (3.7)
	RF & anti-CCP+	13 (8)
	Do not recall	84 (51.9)
Pregnancy Outcomes	Live Births	99 (96)
	Stillbirths	2 (1.9)
	Miscarriages	23 (22.3)
	Abortions	15 (14.5)
Total number of pregnancies per patient	1	29 (27.2)
	2	40 (38.8)
	3	23 (22.3)
	4	6 (5.8)
	5–6	6 (5.8)

IA - inflammatory arthritis, RA - rheumatoid arthritis, PsA - psoriatic arthritis, RF- rheumatoid factor; anti-CCP -anti– cyclic citrullinated peptide antibodies.

Thirty-six percent (36.4%) (59/162) of patients were never pregnant due to social reasons such as financial, relationship status or personal choice (18/59), RA/PsA medications (15/59), infertility (10/59) and RA/PsA disease activity (9/59) (7/59 patients provided no or unclear reason). Of the patients who had pregnancies, 33% of patients had fewer children than desired due to RA/PsA medications (15/34), RA/PsA disease activity (14/34), infertility (5/34), social reasons (5/34) and other co-morbidities (5/34) (Table 2). In comparison to patients who had their desired number of children, patients with fewer children than desired had fewer total pregnancies, live pregnancies, therapeutic abortions and more stillbirths and miscarriages (Table 3).

**Table 2:** Reasons for limiting family size and for not having children.

Reasons	Patients never pregnant (59/162) (# (%))	Patients limiting family size (34/103) (# (%))
RA/PsA medication	15 (25.4)	15 (44.1)
Disease activity	9 (15.3)	14 (41.2)
Infertility	10 (16.9)	5 (14.7)
Social reasons	18 (30.5)	5 (14.7)
Co-morbidities	4 (6.8)	5 (14.7)

**Table 3:** Detailed Pregnancy Outcomes.

	All patients with pregnancies (Mean +/− SD)	Patients with less children than desired (Mean +/− SD)	Patients with expected number of pregnancies (Mean +/− SD)
Total pregnancies	2.27 +/− 1.18	1.97 +/− 1.36	2.42 +/− 1.46
Live pregnancies	1.78 +/− 0.83	1.41 +/− 1.03	1.96 +/− 1.11
Stillbirths	0.03 +/− 0.14	0.03 +/− 0.41	0.02 +/− 0.08
Miscarriages	0.29 +/− 0.64	0.38 +/− 0.56	0.26 +/− 0.45
Therapeutic abortions	0.17 +/− 0.45	0.15 +/− 0.49	0.19 +/− 0.49

In the 63 pregnancies that occurred after their RA/PsA diagnosis (excluding those with therapeutic abortions): (1) pre-conception counseling was obtained in 49% of pregnancies; (2) no statistically significant difference was noted between those with pregnancies who received pre-conception counseling (31/63) and those that did not (32/63) in terms of disease control during pregnancy, discontinuing medication and pregnancy complications including pregnancy induced hypertension, pre-eclampsia/eclampsia, gestational diabetes, multiple pregnancies, IUGR, placental abruption, flare and hospitalizations; (3) most described good disease activity control during pregnancy (41/63 pregnancies, 65%) but flared (28/38, 73.7%) in the first 3 months postpartum (19/28 patients, 67.9%); (4) RA/PsA medication (including NSAIDs, steroids, s/bDMARDs) was discontinued during 79% of pregnancies (Table 4); (6) no statistically significant difference noted in disease control at the time of pregnancy and disease flare during pregnancy between pregnancies with medication discontinuation and those pregnancies who continued medication (7) 35% of pregnancies (22/63) occurred while on biologic therapy at the time of or prior to conception and continued for all or part of the pregnancies in 5 cases (3/5 with complications including ectopic pregnancy, IUGR, multiple birth, flare).

**Table 4:** Peri-partum outcomes in the 63 pregnancies that occurred after their RA/PsA diagnosis (excluding TAs).

	Pregnancies post diagnosis # (%)
Received Pre-conception counseling	31 (49.2)
RA/PsA disease inactive during pregnancy	41 (65.1)
Disease flare post -partum	28 (73.7)
Disease flare in the first 3 months post-partum	19 (67.9)
Discontinued medication	50 (79.4)

For patients planning conception (46/63 post-arthritis, 107/153 pre-arthritis diagnosis pregnancies) time to pregnancy was 0–2 months in 41% post-arthritis vs 46% pre-arthritis diagnosis pregnancies (Table 5). Gestational age at time of delivery was 37–40 weeks in 58% (33/57) post-arthritis vs 66% (83/126) pre-arthritis diagnosis pregnancies. There were no statistically significant differences in pregnancies that occurred before or after diagnosis regarding pregnancy planning, use of fertility treatment (Clomiphene, hCG/FSH/hMG/GnRH agonist/GnRH antagonist hormone injections, IVF and IUI), pregnancy complications (with and without disease flare), labor and delivery complications, delivery methods, breastfeeding, birth defect frequency or neonatal complications. Pregnancy complications (with and without disease flare), labor/delivery complications and C-section deliveries were lower in pregnancies prior to diagnosis; however, this was not statistically significant. Neonatal ICU admissions were significantly lower in pre-arthritis diagnosis pregnancies compared to post-arthritis diagnosis pregnancies. No pregnancy complications were noted in 24/54 pregnancies on medications compared to 6/9 pregnancies not on medications (not statistically different) (Table 5).

**Table 5:** Pregnancy variables in pre-and post-RA/PsA pregnancies.

	# (%) of pregnancies prior to RA/PsA diagnosis	#(%) of pregnancies post RA/PsA diagnosis	P value
Planed pregnancy	107 (69.9)	46 (73.0)	P = 0.65
Time to pregnancy 0–2 months	49 (45.8)	19 (41.3)	P = 0.61
Infertility treatment	13 (8.7)	2 (3.2)	P = 0.24*
37–40 weeks Gestational age at delivery	83 (65.8)	33 (57.9)	P = 0.23
No pregnancy complications	86 (56.2)	30 (47.6)	P = 0.25
Labour and delivery complications	28 (22.2)	15 (26.3)	P = 0.55
C-section Delivery	27 (21.3)	17 (29.8)	P = 0.21
Low birth weight (<2.5 kg)	6 (4.8)	4 (7.0)	P = 0.51*
Breast feeding	96 (76.2)	46 (80.7)	P = 0.50
Birth defects	8 (6.3)	3 (5.5)	P = 1.00*
Neonatal medical complications	19 (15.1)	12 (21.8)	P = 0.27
Neonatal ICU admissions	4 (3.2)	8 (14.5)	P = 0.008

P-values obtained by Chi-square test or Fisher’s exact test (marked as*).

When comparing the 1^st^ pregnancy of patients who had all pregnancies prior to arthritis diagnosis to 1^st^ pregnancy of patients who had all pregnancies after the diagnosis, the patients with 1^st^ pregnancy after arthritis diagnosis were older and had a longer time to achieve pregnancy, although this was not statistically significant. There was also no significant difference in terms of the number of patients who used infertility treatment such as *in vitro* fertilization, intrauterine insemination, and hormone injections as well as pregnancy complications between these 2 groups.

## Discussion

This survey of the Northern Alberta RAPPORT patient participants provides real-world insight into pregnancy outcomes for RA/PsA patients and the careful considerations women must make in contemplating this journey in the context of these diseases. We identified that a third of women with RA/PsA had fewer children than desired due to factors including disease activity and medications. In women with a diagnosis of RA/PsA, flares were very common post-partum. Disease-related medications were discontinued in nearly eighty percent of women during or prior to pregnancy. No difference was found in peripartum outcomes for women who had pregnancies before compared to after their arthritis diagnosis except for neonatal ICU admissions.

This data is important in light of evolving clinical trial and observational data on newer agents in treating RA/PsA and changing opinions of safety of various agents (eg. TNFi). Continued heterogeneity exists in the peri-partum experience amongst women as evidenced by our results, which reflects a complex interplay between patient and rheumatologists and managing RA/PsA during childbearing years.

Our study confirms that patients with RA/PsA who had fewer children than desired had fewer total pregnancies, live pregnancies, therapeutic abortions and more stillbirths, and miscarriages compared to those patients who had expected number of children^12^. This likely represents the true nature of this population as the anonymity of our survey-based study likely lead to limitation of the social desirability bias, as the patient answers were not shared with care providers.

Our study demonstrates that approximately 36% of all the survey respondents were never pregnant mainly due to their arthritis related concerns and infertility. Furthermore, 33% of patients with pregnancies had less children than desired due to their arthritis related concerns and infertility. These results parallel previous studies indicating that arthritis related concerns in addition to infertility lead to smaller family sizes in a substantial number of patients who have not finished having children at the time of the RA/PsA diagnosis^10,12^. Given the availability of pregnancy safe medications for RA/PsA patients in the peripartum period^10,15,16^, the perception of the negative effects of RA/PsA medications seems to be an unnecessary reason for decreased fecundity. Provision of accurate and appropriate education regarding these medications and the impact of pregnancy and disease on each other is essential to ensure patients make informed decisions about reproduction. In contrast to Clowse *et al*.^12^, we found a lower infertility rate among patients who had fewer children than desired, possibly reflecting survey bias whereby patients with difficult reproductive histories may not have completed the survey.

Similar to other studies, we found that the majority of RA/PsA patients reported low disease activity during pregnancy but flare post-partum. Previous studies have shown disease improvement rates between 48%–95%, which is consistent with the low disease activity state of 65% in our study. Furthermore, our postpartum flare rate of 74% was similar to rates of 39–90% demonstrated in previous studies^10,17,18^. The rates observed in our study are on the higher end of the spectrum, likely because subjective measurements were used in our study in keeping with the higher disease activity scores reported in patient reported outcomes compared to physician scores based on objective measurements used in other studies^17^. It is important to note that the rate of remission/improvement during pregnancy is less than what is commonly perceived and given the moderate- high rates of disease flare post-partum^18–20^, continuation of appropriate medication during and after pregnancy is imperative to prevent RA/PsA disease flares.

Our survey identified a statistically increased neonatal ICU admission rate in pregnancies that occurred after the diagnosis of RA/PsA, similar to the findings of Barnabe *et al*.^21^. We speculate that possible contributions to this adverse outcome include use of medications such as corticosteroids, reproductive technologies, and active disease. Although not statistically significant, pregnancies occurring after the diagnosis of IA had more pregnancy complications, labour and delivery complications and C-section deliveries compared to pregnancies occurring before the RA/PsA diagnosis^10^. Age may have been a contributing factor for increased complications as patients with all pregnancies after RA/PsA diagnosis were older compared to patients with all pregnancies prior to diagnosis, although this was not statistically significant in our study, likely due to the low and imbalanced sample size between the two groups. In addition to lower numbers, peri-natal complications may not have achieved statistical significance as the majority (65%) of pregnancies occurred in periods of low disease activity which is associated with better peri-partum outcomes and because of the demographics of the study participants^18–21^.

As expected, due to concerns of teratogenicity, the majority of patients discontinued RA/PsA medications during pregnancy in our study^15^. Our study found no statistically significant difference in disease control during pregnancy between pregnancies with medication discontinuation and those pregnancies who continued medication likely due to the fact that not all medications (i.e s/bDMARDs) were discontinued. Furthermore, if just one medication was discontinued (eg. Steroids, or 1 DMARD if on multiple), these patients were counted under “discontinued medication group” even if the patients may have still been maintained on certain s/bDMARDs. It is also possible that pregnancy may have kept the disease under better control. While the use of certain DMARDs such as methotrexate and leflunomide is restricted during pregnancy and breast-feeding, increasingly more treatment options (eg. hydroxychloroquine, sulfasalazine, TNFi) are available to maintain remission or low disease activity state than ever before^10,15,16^. Despite the data showing low risk of tumor necrosis factor inhibitors (TNFi) use during pregnancy^8,14,16,20,22^, only 5 pregnancies in the study continued biologic therapy and three cases had notable complications, the details of which are not available due to the nature of the survey. Possible explanations include incorrect fear of medication teratogenicity, pregnancies that occurred in the earlier part of the RAPPORT cohort where experience in using TNFis was limited and patient reluctance. Of the 5 pregnancies, only 1 continued biologic therapy into the 3^rd^ trimester.

Several limitations must be noted in this study. Patients included both RA and PsA patients even though these are two distinct disease entities that may confer different risks. Furthermore, the smaller sample size may have led to the lack of significant associations between perinatal events and RA/PsA. This was a retrospective, self-reported survey where quality would be downgraded due to selection and recall bias. Issues surrounding childbearing can be extremely sensitive and our response rate of thirty-seven percent may reflect a reluctance of some women to share their experience but was considered reasonable in comparison to other pregnancy-survey studies^23^. We created responses requiring dichotomous or drop-down list answers to reduce free text and ensure more homogenous data collection.

In conclusion, our study demonstrates that peri-partum complications in RA/PsA patients do not significantly increase in patients with good disease control; however, neonatal ICU admissions are increased in women with RA/PsA revealing the importance of decision-making before, during and after pregnancy. Most patients discontinued RA/PsA medication during pregnancy and despite the data showing low risk of TNFi use during pregnancy, only a minority of patients continued biologics during pregnancy, highlighting the lack of consensus on use and the appropriate time to discontinue TNFi. Therefore, providing patients with medication and disease information is essential to ensure that patients make informed and educated reproductive decisions.

## Supplementary information

Supplemental Appendix 1.
