# Supplementary material for: Peripartum issues in the inflammatory arthritis patient: A survey of the RAPPORT registry
Source: Sci Rep. 2020 Feb 28;10:3733. doi: 10.1038/s41598-020-60451-2 (PMC7048931; doi:10.1038/s41598-020-60451-2)
Supplement: Supplementary file 1 — Supplemental Appendix 1. [file 41598_2020_60451_MOESM1_ESM.pdf]

APPENDIX 1: Peripartum issues in the inflammatory arthritis patient: A survey of the RAPPORT registry

Dissanayake TD, Maksymowych WP, Keeling SO.

Pregnancy Survey

Email ___________________________________

1. What is your current age?
    - < 19
    - 20-30
    - 31-40
    - 41-50

2. Have you been diagnosed with inflammatory arthritis?
    - Yes
    - No
    - If yes, please specify which one:
    - Rheumatoid Arthritis
    - Psoriatic Arthritis

3. What blood antibodies were you positive for?
    - Rheumatoid Factor (RF)
    - Anti-CCP
    - Do not recall

4. What other medical conditions do you have? Please check all that apply:
    - Coronary artery disease (Heart disease)
    - Cerebrovascular disease(Stroke or TIA)
    - Diabetes
    - Hypertension (High blood pressure)
    - Dyslipidemia (High cholesterol/lipids)
    - Thyroid Disease
    - Other
    - Other, please specify ___________________________________
    - None

5. Have you ever been pregnant?
    - Yes
    - No

If you have not ever been pregnant, can you share the reason if you are comfortable?

____

6. If you have previously been pregnant, how many total pregnancies have you had (including live births, stillbirths, miscarriages, therapeutic abortions)?
    - 1
    - 2
    - 3
    - 4
    - 5
    - 6
    - 7
    - 8

9
Over 9

7. If you have previously been pregnant, how many of the following have you had?
   i. Live births? 0,1,2,3,4,5,6,7,8,9,Over 9
   ii. Stillbirths? (Death of a baby at 20 weeks of pregnancy or later, but before birth) 0,1,2,3,4,5,6,7,8,9, Over 9
   iii. Miscarriages? (Loss of a baby before 20 weeks of pregnancy) 0,1,2,3,4,5,6,7,8,9, Over 9
   iv. Therapeutic abortions? 0,1,2,3,4,5,6,7,8,9, Over 9

8. If you had less children than desired, what was the reason for limiting family size? Please check all that apply:
   Sexual dysfunction
   Inflammatory arthritis medications
   Inflammatory arthritis disease activity
   Other; please specify ___________________________________

Please fill out the following sections per pregnancy

Pregnancy 1

1. Which type of pregnancy?
   Live birth
   Stillbirth
   Miscarriage
   Therapeutic abortion

2. What was your age at the time of your pregnancy?
   < 20
   21-25
   26-30
   31-35
   36-40
   41-45
   46-50

3. Were you already diagnosed with Inflammatory Arthritis at the time of your pregnancy?
   Yes
   No

4. What medications were you taking for your Inflammatory Arthritis when you realized you were pregnant? Please check all that applies:
   NAIDS (Anti-Inflammatory eg. Ibuprofen (Advil), Naproxen (Aleve), celecoxib (Celebrex), diclofenac (Arthrotec/Voltaren))
   Prednisone (Steroids)
   Methotrexate
   Leflunomide (Arava)
   Hydroxychloroquine (Plaquenil)
   Sulfasalazine/Salazopyrin
   Etanercept (Enbrel)
   Infliximab (Remicade)
   Adalimumab (Humira)
   Certolizumab (Cimzia)
   Golimumab (Simponi)
   Rituximab (Rituxan)

Tocilizumab (Actemra)
Abatacept (Orencia)
Chloroquine (Aralen)
Tofacitinib (Xeljanz)
Apremilast (Otezla)
Do not recall
Other
Other, please specify ___________________________________
None

5. Did you receive any pre-conception (pre-pregnancy) counseling?
Yes
No

If yes, check all that apply:
Need to control Inflammatory Arthritis symptoms before pregnancy
Need to stop certain Inflammatory Arthritis medications before /during pregnancy
Education that symptoms may improve during pregnancy and flare after delivery
Other
Other, please specify ___________________________________

6. Was your Inflammatory Arthritis under good control(limited pain, tenderness or swelling) at the time you became pregnant?
Yes
No
Do not recall

7. Did you stop any Inflammatory Arthritis medication(s) while you were pregnant?
Yes
No
Do not recall

If yes, check all that apply:
NAIDS (Anti-Inflammatory)
Prednisone (Steroids)
Methotrexate
Leflunomide (Arava)
Hydroxychloroquine (Plaquenil)
Sulfasalazine (Salazopyrin)
Etanercept (Enbrel)
Infliximab (Remicade)
Adalimumab (Humira)
Certolizumab (Cimzia)
Golimumab (Simponi)
Rituximab (Rituxan)
Tocilizumab (Actemra)
Abatacept (Orencia)
Chloroquine (Aralen)
Tofacitinib (Xeljanz)
Apremilast (Otezla)
Do not recall
Other
Other, please specify ___________________________________
None

8. If you were on a biologic therapy (such as infliximab, etanercept, adalimumab, certolizumab,

rituximab, abatacept, tocilizumab) at the time you became pregnant, did you continue your biologic therapy for all or part of your pregnancy?

Yes
No
Not applicable
Do not recall

9. If you were on a biologic therapy during your pregnancy, for how many weeks of pregnancy (eg Gestational age) did you continue the biologic therapy?

Less than 5 weeks pregnancy
5-8 weeks
9-12 weeks
3-16 weeks
17-20 weeks
21-24 weeks
25-27 weeks
28-31 weeks
32-35 weeks
36-37 weeks
Over 37 weeks
Not applicable
Do not recall

Fertility

10. Was your pregnancy planned?

Yes
No

11. If your pregnancy was planned, how long did it take for you to become pregnant?

0-2 months
3-5 months
6-9 months
10-12 months
Over 12 months

12. How long after your Inflammatory Arthritis diagnosis did you get pregnant?

Less than 12 months
1-2 years
3-5 years
5-10 years
Over 10 years
Not applicable
Do not recall

13. Did you require any infertility treatment or medication?

Yes
No

If yes, Please circle all that apply: Consultation with fertility specialist

Clomiphene (Clomid or Serophene)
hCG hormone injections (Novarel, Ovidrel, Pregnyl,Profasi)
FSH hormone injections (Bravelle, Fertinex, Follistim, Gonal-F)
hMG hormone injections (Menpour, Metrodin,Pregonal, Repronex)
GnRH agnosit hormones injections (Lupron, Synarel, Zoladex)
GnRH antagonist hormone injections (antagon, Cetrotide)

IVF (In Vitro Fertilization)
IUI (Intrauterine Insemination)
Do not recall
Other
Other, please specify ___________________________________

Pregnancy History

14. During your pregnancy, did you experience any of the following?
    Pregnancy induced hypertension (High blood pressure after 20 weeks)
    Pre-eclampsia (Toxemia with high blood pressure and protein in your urine)
    Eclampsia (Seizures/convulsions with high blood pressure)
    Gestational Diabetes Mellitus (high blood sugar first diagnosed during pregnancy
    Ectopic pregnancy
    Miscarriage
    Therapeutic Abortion performed by a medical expert
    Multiple Gestations (carrying more than one baby)(eg. twins, triplets)
    Intrauterine growth retardation (IUGR, poor growth of the fetus during pregnancy)
    Placental abruption (placental lining has separated from the uterus)
    Placental previa (placenta partially or fully blocking the neck of the uterus)
    Sepsis/infection during pregnancy
    Flare of your Inflammatory Arthritis
    Hospitalizations
    Do not recall
    None
    Other, please specify: ______________________

If Miscarriage, please specify which trimester:
    1st trimester
    2nd trimester
    3rd trimester

If Therapeutic Abortion performed by a medical expert, please specify which trimester:
    1st trimester
    2nd trimester
    3rd trimester

If Hospitalizations, why? ___________________________________

Delivery

15. What was the method of delivery?
    C/section
    Assisted birth (Instruments - forceps, vacuum extractor)
    Vaginal delivery

16. Did you experience any complications during labor or delivery?
    Yes
    No

If yes, please check all that apply:
    Preterm labour (delivery < 37 weeks)
    Premature rupture of membrane
    Prolonged labour (labor lasting for >20 hours (1stpregnancy) and >14 hours (subsequent pregnancies))

Hemorrhage (bleeding)
Infection
Umbilical cord prolapse (umbilical cord comes out of the uterus with or before the presenting part of the fetus)
Stillbirth (baby born with no signs of life at or after 24 weeks gestation)
Do not recall
Other, please specify ___________________________________

17. How many weeks pregnant were you at the time of delivery?
Less than 24 weeks
24-26 weeks
27-30 weeks
31-34 weeks
35-36 weeks
37-40 weeks
Over 40 weeks
Do not recall

18. How much did your baby weigh at birth?
Less than 4lbs (less than 1.8kg)
4-5.4lbs (1.8- 2.4kg)
5.5-7.5lbs(2.5-3.4kg)
7.6-10lbs(3.45-4.5kg)
Over 10lbs(over 4.5kg)
Do not recall

Post Partum

19. Did you breastfeed post partum?
Yes
No
Not applicable
Do not recall

If yes, indicate how many months in total:
0-3 months
3-6 months
7-9 months
10-12 months
12-18 months
19-24 months
Over 24 months

If no, check all that apply:
Choice
Inflammatory arthritis medications
Inflammatory arthritis disease activity
Do not recall
Other
Other, please specify ___________________________________

20. If you discontinued your Inflammatory Arthritis medications during pregnancy, did you restart any of the medication(s) following delivery of your baby?
Yes
No
Not applicable

Do not recall

If yes, check all that apply:

NAIDS (Anti-Inflammatory)
Prednisone (Steroids
Methotrexate
Leflunomide (Arava)
Hydroxychloroquine (Plaquenil)
Sulfasalazine
Etanercept (Enbrel)
Infliximab (Remicade)
Adalimumab (Humira)
Certolizumab (Cimzia)
Golimumab (Simponi)
Rituximab (Rituxan)
Tocilizumab (Actemra)
Abatacept (Orencia)
Chloroquine (Aralen)
Tofacitinib (Xeljanz)
Apremilast (Otezla)
Do not recall
Other
Other, please specify ___________________________________
None

21. If you restarted your Inflammatory Arthritis medications post partum, how soon did you resume any of the medication(s) following the delivery of your baby?

Within 3 months
3-6 months
7-9 months
10-12 months
12-18 months
19-24 months
over 24 months
Do not recall
Not applicable

22. Did you experience any worsening of your Inflammatory Arthritis symptoms (flare) following delivery?

Yes
No
Do not recall

If yes, indicate how many months after delivery this occurred:

0-3 months
3-6 months
7-9 months
10-12 months
12-18 months
19-24 months
Over 24 months
Do not recall
Not applicable

23. If you experienced a flare post partum, how was this managed in the short-term? Please check all that

apply:

Steroid injections
Anti-inflammatory (NSAIDs)
Prednisone (Oral steroid)
Pain killers
Rest
Exercise
Other, please specify ___________________________________

24. Did you start any new Inflammatory Arthritis medications post-partum?

Yes
No

If yes, check all that apply:

NAIDS (Anti-Inflammatory)
Prednisone (Steroids
Methotrexate
Leflunomide (Arava)
Hydroxychloroquine (Plaquenil)
Sulfasalazine
Etanercept (Enbrel)
Infliximab (Remicade)
Adalimumab (Humira)
Certolizumab (Cimzia)
Golimumab (Simponi)
Rituximab (Rituxan)
Tocilizumab (Actemra)
Abatacept (Orencia)
Chloroquine (Aralen)
Tofacitinib (Xeljanz)
Apremilast (Otezla)
Do not recall
Other, please specify ___________________________________
None

25. If you started any new Inflammatory Arthritis medications, when did you start them following delivery?

Within 3 months
3-6 months
7-9 months
10-12 months
12-18 months
19-24 months
Over 24 months
Do not recall
Not applicable

Neonatal complications

26. Did your baby have any birth defects?

Yes
No
Do not recall

If yes, check all that apply:
Limb defect

Heart defect
Intestinal defect
Spina bifida
Other, please specify ___________________________________
Cleft lip/palate

27. Did the baby have any medical complications post partum (within 3 months of delivery)?
    Yes
    No
    Do not recall

    If yes, check all that apply: Infection
    Bleeding
    Breathing difficulty
    Feeding difficulty
    Jaundice
    Other
    Other, please specify ___________________________________

28. Did the baby require admission to the intensive care unit?
    Yes
    No
    If yes, specify reason and duration: ___________________________________
